# Crystal structure of a mixed-ligand silver(I) complex of the non-steroidal anti-inflammatory drug diclofenac and pyrimidine

**DOI:** 10.1107/S2056989016014730

**Published:** 2016-09-27

**Authors:** Sevim Hamamci Alisir, Necmi Dege

**Affiliations:** a Department of Materials Science and Engineering, Faculty of Engineering, Ondokuz Mayis University, Samsun 55139, Turkey; b Department of Physics, Faculty of Arts and Sciences, Ondokuz Mayis University, Samsun 55139, Turkey

**Keywords:** crystal structure, silver(I) complex, non-steroidal anti-inflammatory drug, diclofenac, two-dimensional coordination polymer

## Abstract

In the title mixed-ligand silver(I) coordination polymeric complex with the non-steroidal anti-inflammatory drug diclofenac (C_14_H_11_Cl_2_NO_2_) (diclH) and pyrimidine (pym), namely poly[{μ_2_-2-[2-(2,6-di­chloro­anilino)phen­yl]acetato-κ^2^ *O*:*O*′}(μ_2_-pyrimidine-κ^2^ *N* ^1^:*N* ^3^)silver(I)], [Ag(C_14_H_10_Cl_2_NO_2_)(C_4_H_4_N_2_)]_*n*_ or [Ag(μ-dicl)(μ-pym)]_*n*_, the very distorted tetra­hedral AgN_2_O_2_ coordination centres comprise two N-atom donors from bridging pym ligands [Ag—N = 2.381 (3) and 2.412 (3) Å] and two carboxyl­ate O-atom donors from dicl ligands [Ag—O = 2.279 (2) and 2.280 (2) Å], which bridge Ag atoms, giving a centrosymmetric dinuclear units with a short Ag⋯Ag separation [2.8931 (5) Å]. Within the units are short intra­ligand C—Cl⋯π(pym) inter­actions [3.6409 (15) Å]. The units are linked through the bridging N atoms of the pym ligand into a two-dimensional sheet–polymer structure lying parallel to (100) and stabilized by inter-ring π–π inter­actions between the pym ligands [*Cg*⋯*Cg* = 3.4199 (17) Å]. Additional inter-unit C—H⋯O and C—H⋯*Cg* hydrogen-bonding inter­actions between the sheets give an overall three-dimensional structure.

## Abstract 2

The coordination polymeric silver(I)–diclofenac complex including pyrimidine is based on a centrosymmetric carboxyl­ate *O*:*O′*-bridged dinuclear unit which is extended through N-atom donors of the pyrimidine ligand into a two-dimensional layered structure

## Chemical context   

The design of coordination polymers based on silver(I) has been studied extensively in recent years because of their various structural topologies as well as photoluminescent properties and anti­microbial activity. These studies have shown that short Ag⋯Ag separations are one of the most important factors for the manifestation of such properties [Yam & Lo, 1999 ▸; Pyykkö *et al.*, 1997 ▸; Wang & Cohen, 2009 ▸; Zhang *et al.*, 2009 ▸, Njogu *et al.*, 2015 ▸; Nomiya *et al.*, 2000 ▸]. On the other hand, it is known that to construct extended coordination networks with polynuclear metal-based structures, ligands of various binding sites and shapes have to be taken into account. At this stage, confidence in accomplishing this goal is based upon the sophisticated selection and utilization of suitable multifunctional organic ligands with certain features, such as being a multiple donor and having versatile bonding modes or the ability to take part in hydrogen bonding. Aromatic carboxyl­ate derivatives have therefore been of inter­est in coordination and supra­molecular chemistry.

The chemical classes of non-steroidal anti-inflammatory drugs (NSAIDs) consist of salicylate derivatives, phenyl­alkanoic acids, oxicams, anthranilic acids, sulfonamides and furan­ones (Weder *et al.*, 2002 ▸). These compounds are some of the most commonly used medications to reduce pain, and diclofenac (dicl), [2-(2,6-dicholoroanilino)phenyl­acetic acid], is a member of the group of phenyl­alkanoic acids. Additionally, NSAIDs are used as anti-inflammatories, anti­pyretics and anti­tumor drugs. (Kim *et al.*, 2004 ▸; Ribeiro *et al.*, 2008 ▸; Duffy *et al.*, 1998 ▸). In previous publications, the crystal structures of metal complexes of diclofenac have been reported (Caglar *et al.*, 2013 ▸, 2014 ▸; Ali & Jabali, 2016 ▸; Dimiza *et al.*, 2011 ▸; Kovala-Demertzi *et al.*, 1997 ▸; Castellari *et al.*, 1999 ▸; Kourkoumelis *et al.*, 2004 ▸) and in addition its mol­ecular structure has been characterized by various techniques (Iliescu *et al.*, 2004 ▸). Based on the above-mentioned points, we report herein the synthesis and structural characterization of a new mixed-ligand silver(I) complex with dicl and pyrimidine (pym), namely [Ag(μ-dicl)(μ-pym)]_*n*_, (I).
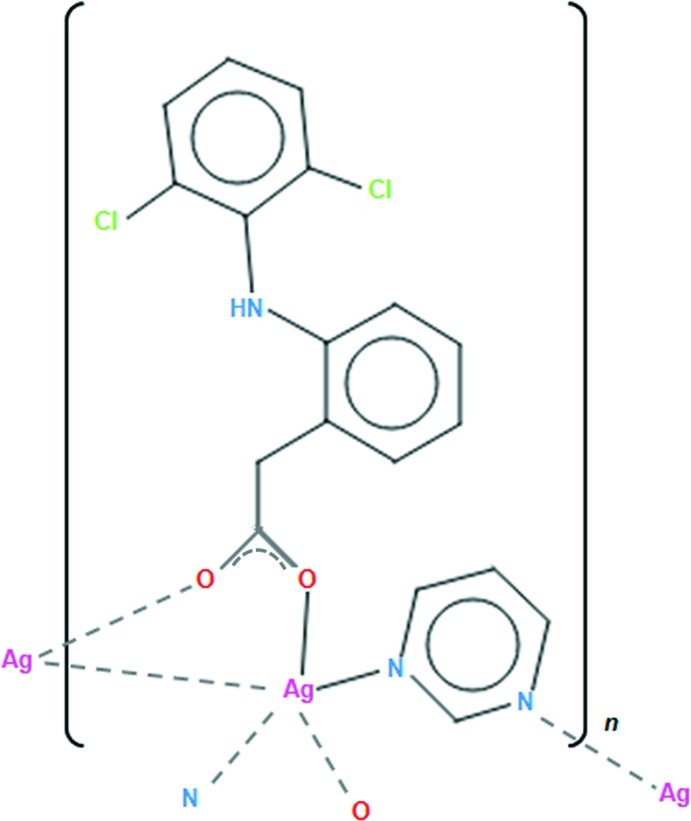


## Structural commentary   

**Figure 1:**
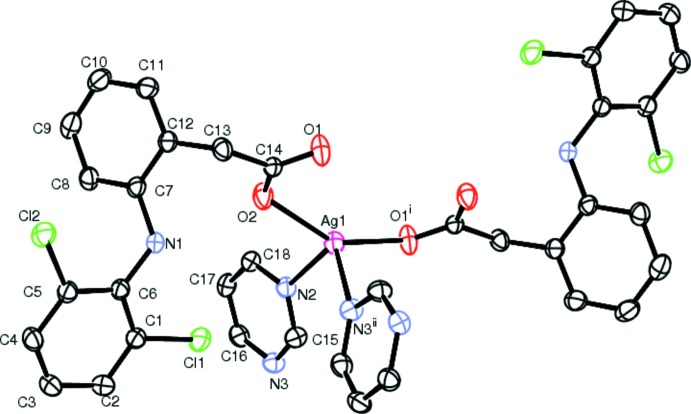
The mol­ecular configuration and atom-labelling scheme for the title complex, (I), with displacement ellipsoids drawn at the 30% level. For symmetry codes (i) and (ii), see Table 1 ▸.

**Figure 2:**
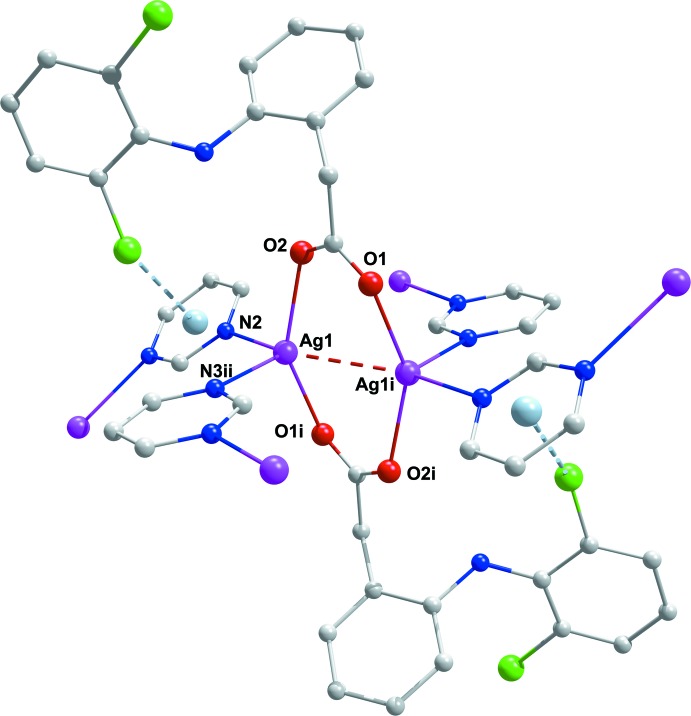
A view of the centrosymmetric caboxylate-bridged dinuclear [Ag_2_(μ-dicl)_2_] unit in (I). H atoms have been omitted.

In (I), Ag1 atoms are four-coordinated by two carboxyl­ate oxygen atoms [O2 and O1^i^; symmetry code: (i) −*x* + 1, −*y* + 1, −*z* + 2] from separate dicl ligands and two nitro­gen atoms [N2 and N3^ii^; symmetry code: (ii) *x*, −*y* + 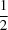, −*z* + 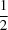] from two separate pym ligands (Fig. 1 ▸). The discrimination parameter for the AgN_2_O_2_ core {τ_4_ = [(360° − (α + β)]/141°}, where α and β are the largest angles around the metal atom) is 0.732 and indicates substantial deviation from ideal tetra­hedral geometry (Yang *et al.*, 2007 ▸). The Ag—N bond lengths [2.381 (3) and 2.412 (3) Å] (Table 1 ▸) are similar to those found in the polymeric mixed-ligand silver(I) complex with 3,5-pyridinedi­carboxyl­ate (pydc) and (pym), [Ag_4_(μ-pydc)_2_(μ-pym)_2_]_*n*_ [2.313 (5), 2.436 (5) and 2.490 (5) Å; Hamamci Alisir *et al.*, 2015 ▸). The Ag—O bond lengths in (I) [2.279 (2) and 2.280 (2) Å] are longer than those in [Ag_2_(sal)_2_]_*n*_ (sal = salicylate; 2.1887–2.2043 Å; Azócar *et al.*, 2013 ▸) but shorter than those found in other silver carboxyl­ate complexes (Wu & Mak, 1995 ▸; Zhang *et al.*, 2015 ▸; Olson *et al.*, 2006 ▸). Each pair of silver(I) atoms in the title complex is bridged by the μ_2_-carboxyl­ato-*O,O′* groups of dicl, forming centrosymmetric dinuclear [Ag_2_(μ-dicl)_2_] units (Fig. 2 ▸). Within the units are short intra­ligand C1—Cl1⋯π inter­actions to the pym ligands [3.6409 (15) Å]. The Ag1⋯Ag1^i^ separation in the unit [2.8931 (5) Å] is significantly shorter than the sum of the van der Waals radii for two silver atoms (3.44 Å), indicating weak inter­actions between adjacent Ag^I^ ions, forming an [Ag_2_(COO)_2_] units. If coexisting strong argentophilic Ag1⋯Ag1^i^ inter­actions are considered as coordinative, it could be reasoned that the coordination around Ag1 is slightly distorted trigonal–bipyramidal [the structural distortion index tau (τ) was calculated to be 0.06] (Addison *et al.*, 1984 ▸).

**Figure 3:**
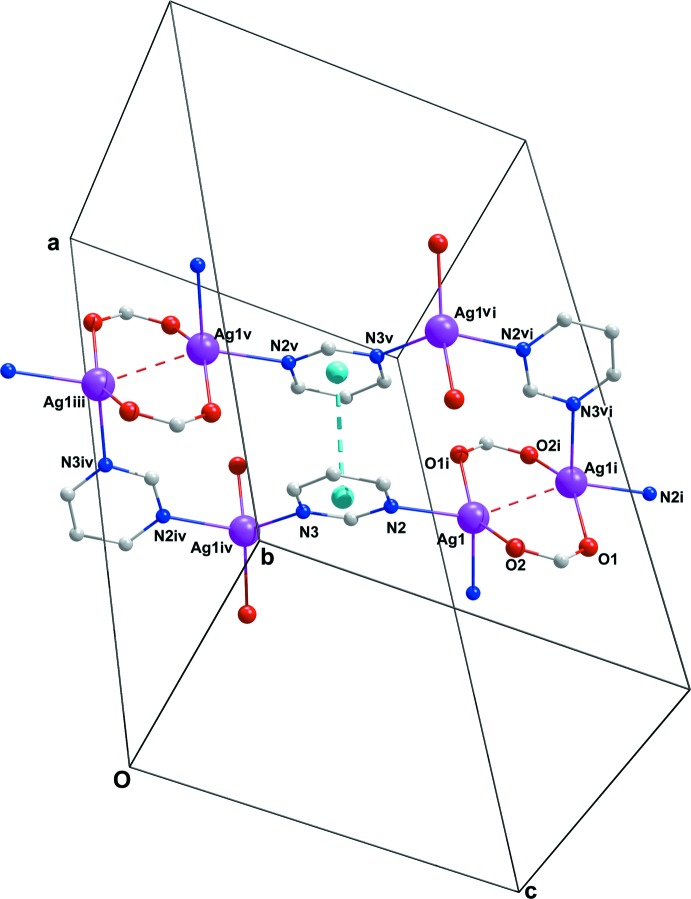
A partial expansion of the dinuclear unit in (I) through the pym ligands, also showing the pym⋯pym π–π ring inter­actions.

**Figure 4:**
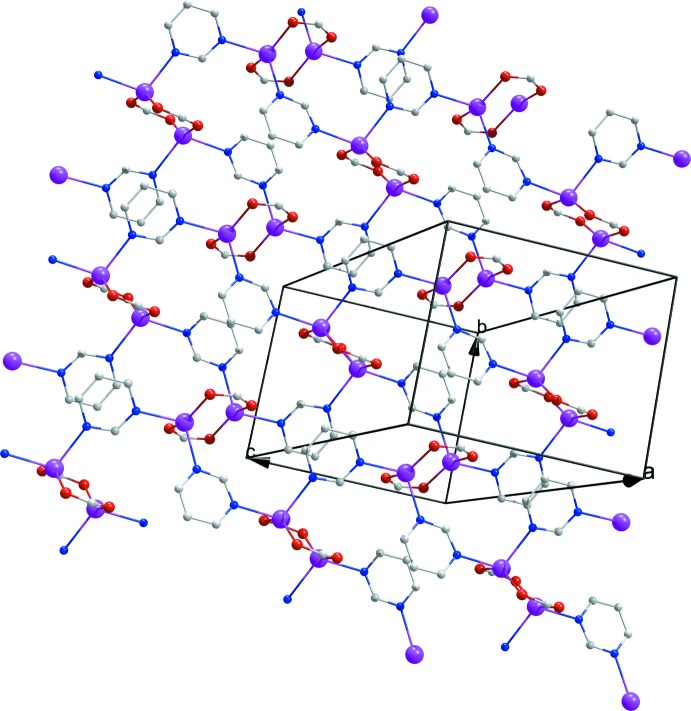
The layered structure of (I). H atoms and part of the dicl ligands have been omitted.

As illustrated in Fig. 3 ▸, in the title complex, the pym ligand acts as a μ_2_-*N,N^1^*-bridging ligand between neighboring [Ag_2_(COO)_2_] units, leading to the formation of a two-dimensional coordination polymer, extending along (100) (Fig. 4 ▸). In other words, [Ag_2_(COO)_2_] units, which comprise eight-membered rings, can be defined as the nodes of the structure. Connection of the four different pym ligands to these nodes provides continuity of the structure (Fig. 4 ▸).

In the dicl ligand, the two benzene rings form a dihedral angle of 61.42 (5)°, the conformation of the ligand being stabilized by an intra­molecular N1—H1⋯O2_carbox­yl_ hydrogen-bonding inter­action [2.971 (3) Å] (Table 2 ▸).

## Supra­molecular features   

**Figure 5:**
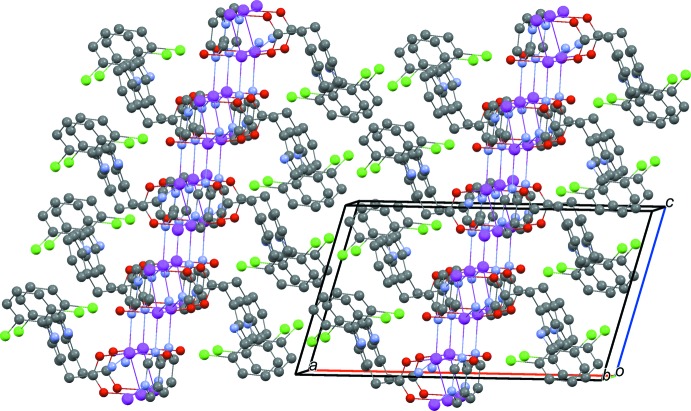
The packing of (I) in the unit cell viewed along the *b* axis.

In the crystal, a C16—H16⋯O1^iii^ hydrogen-bonding inter­action stabilizes the crystal packing (Table 2 ▸). In addition, there is a weak C13—H13⋯*Cg*6^iv^ inter­action to a pym ring [3.983 Å] and a strong π–π stacking inter­action between aromatic rings of the pym ligands [*Cg*3⋯*Cg*3^v^ = 3.4199 (17) Å; *Cg*3 is the centroid of the N2/C15/N3/C216–C18 ring; symmetry code (v): −*x* + 1, −*y* + 1, −*z* + 1], shown in Fig. 3 ▸. These inter­actions are significant for holding layers together in the solid state and generating an overall three-dimensional framework structure (Fig. 5 ▸).

## Synthesis and crystallization   

All reactions were performed with commercially available reagents and used without further purification. Solid sodium 2-(2,6-dicholoroanilino)phenyl­acetate (Nadicl) (0.32 g, 1 mmol) and pyrimidine (0.08 g, 1 mol) were added to an aqueous solution (10 cm^3^) of AgNO_3_ (0.17 g, 1 mmol) with stirring. A white suspension with a white precipitate formed and the addition of aceto­nitrile (10 cm^3^) to this resulted in a clear solution which was left to stand for slow evaporation in darkness at room temperature. Single crystals of (I) suitable for X-ray analysis were obtained within a few days.

## Spectroscopy   

The infrared spectrum was obtained using a Perkin Elmer Spectrum Two FTIR with a diamond Attenuated Total Reflectance attachment (ATR) in the frequency range 4000–600 cm^−1^. The sample was placed on the ATR crystal and pressure exerted by screwing the pressure clamp onto the sample to ensure maximum contact with the ATR crystal. The characteristic absorption bands of Nadicl and the title complex are listed in Table 3 ▸. The spectrum is deposited as a supplementary Fig. S1.

The characteristic absorption band in the FT–IR spectra of the carboxyl­ate complexes is the asymmetric (υ_as_) and symmetric (υ_s_) vibrations of the carboxyl­ate group. The difference between the asymmetric and symmetric carboxyl­ate stretching [Δν = υ_as_(COO^−^) - υ_s_(COO^−^)] is often used to correlate the infrared spectra of metal carboxyl­ate structures. When Δν < 200 cm^−1^, the carboxyl­ate groups of the complexes can be considered bidentate (Azócar *et al.*, 2013 ▸). The value of Δν is calculated as 183 cm^−1^ for 1. Based on the above-mentioned points, it is suggested that carboxyl­ate groups in the complex exhibit a bidentate coordination mode, as revealed by the structural analysis.

## Refinement   

Crystal data, data collection and structure refinement details are summarized in Table 4 ▸. All C-bound hydrogen atoms in (I) were included in calculated positions with C—H = 0.93 Å (aromatic) or 0.97 Å (methyl­ene) and allowed to ride, with *U*_iso_(H) = 1.2*U*_eq_(C). The N-bound H atom was located in a difference-Fourier map but was also allowed to ride in the refinement with *U*_iso_(H) = 1.2*U*_eq_(N).

## Supplementary Material

Crystal structure: contains datablock(s) I. DOI: 10.1107/S2056989016014730/zs2370sup1.cif

Structure factors: contains datablock(s) I. DOI: 10.1107/S2056989016014730/zs2370Isup2.hkl

Supporting information file. DOI: 10.1107/S2056989016014730/zs2370sup3.tif

CCDC reference: 1500646

Additional supporting information: crystallographic information; 3D view; checkCIF report

## Figures and Tables

**Table 1:** Selected geometric parameters (Å, °)

Ag1—O2	2.279 (2)	Ag1—N3^ii^	2.412 (3)
Ag1—O1^i^	2.280 (2)	Ag1—Ag1^i^	2.8931 (5)
Ag1—N2	2.381 (3)		
			
O2—Ag1—O1^i^	148.04 (10)	O2—Ag1—Ag1^i^	81.70 (6)
O2—Ag1—N2	99.71 (8)	O1^i^—Ag1—Ag1^i^	76.19 (6)
O1^i^—Ag1—N2	89.58 (8)	N2—Ag1—Ag1^i^	151.80 (6)
O1^i^—Ag1—N3^ii^	108.69 (9)	N3^ii^—Ag1—Ag1^i^	99.73 (6)
N2—Ag1—N3^ii^	107.93 (9)		

Symmetry codes: (i) 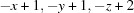; (ii) 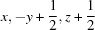.

**Table 2:** Hydrogen-bond geometry (Å, °)

*D*—H⋯*A*	*D*—H	H⋯*A*	*D*⋯*A*	*D*—H⋯*A*
N1—H1⋯O2	0.86	2.43	2.971 (3)	122
C16—H16⋯O1^iii^	0.93	2.51	3.248 (4)	136
C13—H13*B*⋯*Cg*6^iv^	0.97	3.30	3.983 (3)	129

Symmetry codes: (iii) 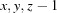; (iv) 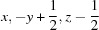.

**Table 3:** Selected comparative IR spectral data for Nadicl and the dicl ligand in (I) Frequencies in cm^−1^; *w*, weak; *m*, medium; *s*, strong; *vs*, very strong. Nadicl = sodium 2-(2,6-di­chloro­anilino)phenyl­acetate.

Assignment	Nadicl	(I)	
ν(NH)	3250 (*m*)	3307 (*m*)	
ν_ar_(CH)	3060 (*vw*)	3064–3029 (*vw*)	
ν_al_(CH)	2980 (*vw*)	2956–2890 (*vw*)	
ν_as_(COO)	1572 (*vs*)	1548 (*vs*)	
ν_s_(COO)	1399 (*w*)	1365 (*vs*)	
ν(CCl)	768 (*s*)	768 (*vs*)	

**Table 4:** Experimental details

Crystal data	
Chemical formula	[Ag(C_14_H_10_Cl_2_NO_2_)(C_4_H_4_N_2_)]
*M*_r_	483.09
Crystal system, space group	Monoclinic, *P*2_1_/*c*
Temperature (K)	293
*a*, *b*, *c* (Å)	18.5886 (4), 9.3071 (4), 10.6646 (8)
β (°)	105.644 (3)
*V* (Å^3^)	1776.69 (16)
*Z*	4
Radiation type	Mo *K*α
μ (mm^−1^)	1.45
Crystal size (mm)	0.60 × 0.46 × 0.27

Data collection	
Diffractometer	Stoe *IPDS2*
Absorption correction	Integration (*X-RED32*; Stoe & Cie, 2002 ▸)
*T*_min_, *T*_max_	0.471, 0.693
No. of measured, independent and observed [*I* > 2σ(*I*)] reflections	13090, 4538, 3672
*R*_int_	0.088
(sin θ/λ)_max_ (Å^−1^)	0.675

Refinement	
*R*[*F* ^2^ > 2σ(*F* ^2^)], *wR*(*F* ^2^), *S*	0.039, 0.095, 1.04
No. of reflections	4538
No. of parameters	236
H-atom treatment	H-atom parameters constrained
Δρ_max_, Δρ_min_ (e Å^−3^)	0.57, −1.14

Computer programs: *X-AREA* and *X-RED32* (Stoe & Cie, 2002 ▸), *SHELXS97* (Sheldrick, 2008 ▸) within *WinGX* (Farrugia, 2012 ▸), *SHELXL2014* (Sheldrick, 2015 ▸), *ORTEP-3 for Windows* (Farrugia, 2012 ▸) and *SHELXTL* (Sheldrick, 2008 ▸).

## supplementary crystallographic information

### Crystal data

**Table:**

[Ag(C_14_H_10_Cl_2_NO_2_)(C_4_H_4_N_2_)]	*F*(000) = 960
*M**_r_* = 483.09	*D*_x_ = 1.806 Mg m^−^^3^
Monoclinic, *P*2_1_/*c*	Mo *K*α radiation, λ = 0.71073 Å
*a* = 18.5886 (4) Å	Cell parameters from 13681 reflections
*b* = 9.3071 (4) Å	θ = 2.0–29.1°
*c* = 10.6646 (8) Å	µ = 1.45 mm^−^^1^
β = 105.644 (3)°	*T* = 293 K
*V* = 1776.69 (16) Å^3^	Prism, colorless
*Z* = 4	0.60 × 0.46 × 0.27 mm

### Data collection

**Table:**

Stoe IPDS2 diffractometer	3672 reflections with *I* > 2σ(*I*)
ω–scan rotation method	*R*_int_ = 0.088
Absorption correction: integration (X-RED32; Stoe & Cie, 2002)	θ_max_ = 28.7°, θ_min_ = 2.3°
*T*_min_ = 0.471, *T*_max_ = 0.693	*h* = −24→24
13090 measured reflections	*k* = −12→12
4538 independent reflections	*l* = −14→14

### Refinement

**Table:**

Refinement on *F*^2^	Hydrogen site location: inferred from neighbouring sites
Least-squares matrix: full	H-atom parameters constrained
*R*[*F*^2^ > 2σ(*F*^2^)] = 0.039	*w* = 1/[σ^2^(*F*_o_^2^) + (0.0488*P*)^2^] where *P* = (*F*_o_^2^ + 2*F*_c_^2^)/3
*wR*(*F*^2^) = 0.095	(Δ/σ)_max_ = 0.001
*S* = 1.04	Δρ_max_ = 0.57 e Å^−^^3^
4538 reflections	Δρ_min_ = −1.14 e Å^−^^3^
236 parameters	Extinction correction: SHELXL2014 (Sheldrick, 2015), Fc^*^=kFc[1+0.001xFc^2^λ^3^/sin(2θ)]^-1/4^
0 restraints	Extinction coefficient: 0.0206 (11)

### Special details

**Table:**

Geometry. All esds (except the esd in the dihedral angle between two l.s. planes) are estimated using the full covariance matrix. The cell esds are taken into account individually in the estimation of esds in distances, angles and torsion angles; correlations between esds in cell parameters are only used when they are defined by crystal symmetry. An approximate (isotropic) treatment of cell esds is used for estimating esds involving l.s. planes.

### Fractional atomic coordinates and isotropic or equivalent isotropic displacement parameters (Å^2^)

**Table:**

	*x*	*y*	*z*	*U*_iso_*/*U*_eq_	
C1	0.17222 (14)	0.3200 (3)	0.6246 (3)	0.0419 (6)	
C2	0.12206 (16)	0.2364 (3)	0.5353 (3)	0.0496 (7)	
H2	0.1387	0.1695	0.4847	0.060*	
C3	0.04629 (16)	0.2541 (4)	0.5223 (4)	0.0546 (8)	
H3	0.0116	0.2009	0.4607	0.065*	
C4	0.02263 (15)	0.3495 (4)	0.5995 (3)	0.0505 (7)	
H4	−0.0282	0.3603	0.5912	0.061*	
C5	0.07381 (14)	0.4302 (3)	0.6904 (3)	0.0406 (6)	
C6	0.15110 (13)	0.4220 (3)	0.7029 (3)	0.0381 (5)	
C7	0.19762 (12)	0.6542 (3)	0.8068 (3)	0.0377 (5)	
C8	0.15520 (14)	0.7377 (3)	0.7067 (3)	0.0451 (6)	
H8	0.1312	0.6953	0.6275	0.054*	
C9	0.14820 (16)	0.8839 (4)	0.7235 (4)	0.0529 (8)	
H9	0.1197	0.9397	0.6558	0.063*	
C10	0.18355 (17)	0.9463 (3)	0.8408 (4)	0.0573 (9)	
H10	0.1785	1.0443	0.8535	0.069*	
C11	0.22657 (15)	0.8624 (3)	0.9396 (4)	0.0501 (7)	
H11	0.2502	0.9054	1.0187	0.060*	
C12	0.23575 (12)	0.7165 (3)	0.9249 (3)	0.0391 (6)	
C13	0.28437 (13)	0.6294 (4)	1.0340 (3)	0.0437 (6)	
H13A	0.2575	0.5429	1.0448	0.052*	
H13B	0.2930	0.6843	1.1140	0.052*	
C14	0.36042 (13)	0.5857 (3)	1.0147 (3)	0.0367 (5)	
C15	0.44153 (16)	0.3069 (3)	0.5812 (3)	0.0444 (6)	
H15	0.4577	0.2251	0.6310	0.053*	
C16	0.40626 (15)	0.4123 (3)	0.3828 (3)	0.0461 (6)	
H16	0.3968	0.4080	0.2926	0.055*	
C17	0.39630 (16)	0.5399 (4)	0.4394 (3)	0.0489 (7)	
H17	0.3807	0.6220	0.3899	0.059*	
C18	0.41040 (15)	0.5417 (3)	0.5740 (3)	0.0461 (6)	
H18	0.4038	0.6266	0.6154	0.055*	
Ag1	0.46373 (2)	0.43353 (3)	0.87652 (2)	0.04354 (10)	
Cl1	0.26694 (4)	0.29612 (9)	0.63922 (10)	0.0610 (2)	
Cl2	0.03984 (4)	0.54045 (9)	0.79226 (9)	0.05280 (19)	
N1	0.20327 (12)	0.5025 (3)	0.7931 (3)	0.0443 (5)	
H1	0.2409	0.4588	0.8434	0.053*	
N2	0.43318 (12)	0.4245 (3)	0.6450 (2)	0.0425 (5)	
N3	0.42916 (13)	0.2935 (3)	0.4527 (2)	0.0457 (5)	
O1	0.41252 (11)	0.5735 (3)	1.1157 (2)	0.0586 (6)	
O2	0.36494 (11)	0.5641 (3)	0.9022 (2)	0.0590 (6)	

### Atomic displacement parameters (Å^2^)

**Table:**

	*U*^11^	*U*^22^	*U*^33^	*U*^12^	*U*^13^	*U*^23^
C1	0.0374 (11)	0.0372 (13)	0.0497 (15)	0.0009 (10)	0.0092 (11)	0.0018 (13)
C2	0.0522 (15)	0.0425 (15)	0.0518 (17)	−0.0023 (12)	0.0097 (13)	−0.0065 (14)
C3	0.0458 (14)	0.0469 (16)	0.063 (2)	−0.0076 (12)	0.0001 (13)	−0.0057 (16)
C4	0.0350 (12)	0.0496 (17)	0.062 (2)	−0.0056 (11)	0.0045 (12)	0.0027 (15)
C5	0.0362 (11)	0.0378 (13)	0.0472 (15)	0.0012 (10)	0.0104 (11)	0.0046 (12)
C6	0.0341 (10)	0.0344 (12)	0.0430 (14)	0.0025 (9)	0.0055 (10)	0.0047 (11)
C7	0.0293 (10)	0.0376 (13)	0.0472 (14)	0.0020 (9)	0.0119 (10)	0.0004 (12)
C8	0.0339 (11)	0.0495 (15)	0.0504 (16)	0.0006 (11)	0.0090 (11)	0.0030 (14)
C9	0.0402 (13)	0.0480 (16)	0.072 (2)	0.0062 (12)	0.0182 (14)	0.0166 (17)
C10	0.0468 (15)	0.0384 (15)	0.089 (3)	0.0036 (12)	0.0230 (16)	−0.0026 (17)
C11	0.0383 (12)	0.0468 (16)	0.067 (2)	−0.0010 (11)	0.0167 (13)	−0.0129 (15)
C12	0.0267 (10)	0.0422 (14)	0.0502 (15)	0.0005 (9)	0.0135 (10)	−0.0046 (12)
C13	0.0319 (11)	0.0561 (17)	0.0432 (15)	0.0005 (11)	0.0102 (10)	−0.0061 (14)
C14	0.0303 (10)	0.0338 (12)	0.0445 (14)	0.0008 (9)	0.0074 (10)	−0.0019 (11)
C15	0.0539 (14)	0.0419 (14)	0.0395 (14)	0.0020 (12)	0.0159 (12)	0.0026 (13)
C16	0.0441 (13)	0.0577 (18)	0.0356 (13)	−0.0024 (12)	0.0094 (11)	0.0018 (13)
C17	0.0451 (13)	0.0503 (16)	0.0493 (16)	0.0086 (12)	0.0096 (12)	0.0110 (14)
C18	0.0404 (12)	0.0432 (15)	0.0522 (17)	0.0056 (11)	0.0084 (12)	−0.0035 (13)
Ag1	0.04020 (13)	0.05477 (15)	0.03712 (13)	0.00513 (8)	0.01297 (8)	−0.00042 (10)
Cl1	0.0401 (3)	0.0555 (4)	0.0877 (6)	0.0068 (3)	0.0176 (3)	−0.0087 (5)
Cl2	0.0458 (3)	0.0534 (4)	0.0647 (5)	0.0013 (3)	0.0243 (3)	−0.0020 (4)
N1	0.0344 (10)	0.0402 (12)	0.0504 (14)	0.0065 (9)	−0.0019 (9)	−0.0026 (11)
N2	0.0401 (10)	0.0500 (14)	0.0375 (11)	0.0031 (9)	0.0104 (9)	−0.0026 (11)
N3	0.0534 (13)	0.0460 (13)	0.0392 (12)	−0.0014 (10)	0.0149 (10)	−0.0015 (11)
O1	0.0334 (9)	0.0967 (19)	0.0426 (11)	0.0109 (10)	0.0049 (8)	0.0071 (12)
O2	0.0412 (10)	0.0880 (18)	0.0456 (12)	0.0186 (10)	0.0080 (9)	−0.0133 (12)

### Geometric parameters (Å, º)

**Table:**

C1—C2	1.379 (4)	C12—C13	1.504 (4)
C1—C6	1.390 (4)	C13—C14	1.537 (3)
C1—Cl1	1.739 (3)	C13—H13A	0.9700
C2—C3	1.387 (4)	C13—H13B	0.9700
C2—H2	0.9300	C14—O2	1.242 (4)
C3—C4	1.362 (5)	C14—O1	1.244 (3)
C3—H3	0.9300	C15—N2	1.320 (4)
C4—C5	1.382 (4)	C15—N3	1.333 (4)
C4—H4	0.9300	C15—H15	0.9300
C5—C6	1.409 (3)	C16—N3	1.338 (4)
C5—Cl2	1.733 (3)	C16—C17	1.367 (5)
C6—N1	1.387 (3)	C16—H16	0.9300
C7—C8	1.382 (4)	C17—C18	1.388 (5)
C7—C12	1.394 (4)	C17—H17	0.9300
C7—N1	1.426 (4)	C18—N2	1.330 (4)
C8—C9	1.383 (5)	C18—H18	0.9300
C8—H8	0.9300	Ag1—O2	2.279 (2)
C9—C10	1.375 (6)	Ag1—O1^i^	2.280 (2)
C9—H9	0.9300	Ag1—N2	2.381 (3)
C10—C11	1.380 (5)	Ag1—N3^ii^	2.412 (3)
C10—H10	0.9300	Ag1—Ag1^i^	2.8931 (5)
C11—C12	1.383 (4)	N1—H1	0.8600
C11—H11	0.9300		
			
C2—C1—C6	123.5 (2)	C12—C13—H13B	108.5
C2—C1—Cl1	118.0 (2)	C14—C13—H13B	108.5
C6—C1—Cl1	118.5 (2)	H13A—C13—H13B	107.5
C1—C2—C3	118.8 (3)	O2—C14—O1	125.6 (2)
C1—C2—H2	120.6	O2—C14—C13	118.5 (2)
C3—C2—H2	120.6	O1—C14—C13	115.9 (3)
C4—C3—C2	120.1 (3)	N2—C15—N3	126.6 (3)
C4—C3—H3	119.9	N2—C15—H15	116.7
C2—C3—H3	119.9	N3—C15—H15	116.7
C3—C4—C5	120.3 (3)	N3—C16—C17	122.1 (3)
C3—C4—H4	119.9	N3—C16—H16	118.9
C5—C4—H4	119.9	C17—C16—H16	118.9
C4—C5—C6	122.0 (3)	C16—C17—C18	117.2 (3)
C4—C5—Cl2	117.6 (2)	C16—C17—H17	121.4
C6—C5—Cl2	120.5 (2)	C18—C17—H17	121.4
N1—C6—C1	121.9 (2)	N2—C18—C17	121.4 (3)
N1—C6—C5	122.8 (3)	N2—C18—H18	119.3
C1—C6—C5	115.2 (2)	C17—C18—H18	119.3
C8—C7—C12	120.6 (3)	O2—Ag1—O1^i^	148.04 (10)
C8—C7—N1	121.4 (3)	O2—Ag1—N2	99.71 (8)
C12—C7—N1	118.1 (2)	O1^i^—Ag1—N2	89.58 (8)
C7—C8—C9	120.5 (3)	O2—Ag1—N3^ii^	97.48 (9)
C7—C8—H8	119.8	O1^i^—Ag1—N3^ii^	108.69 (9)
C9—C8—H8	119.8	N2—Ag1—N3^ii^	107.93 (9)
C10—C9—C8	119.7 (3)	O2—Ag1—Ag1^i^	81.70 (6)
C10—C9—H9	120.2	O1^i^—Ag1—Ag1^i^	76.19 (6)
C8—C9—H9	120.2	N2—Ag1—Ag1^i^	151.80 (6)
C9—C10—C11	119.4 (3)	N3^ii^—Ag1—Ag1^i^	99.73 (6)
C9—C10—H10	120.3	C6—N1—C7	123.3 (2)
C11—C10—H10	120.3	C6—N1—H1	118.4
C10—C11—C12	122.2 (3)	C7—N1—H1	118.4
C10—C11—H11	118.9	C15—N2—C18	116.7 (3)
C12—C11—H11	118.9	C15—N2—Ag1	122.4 (2)
C11—C12—C7	117.6 (3)	C18—N2—Ag1	120.8 (2)
C11—C12—C13	120.6 (3)	C15—N3—C16	115.9 (3)
C7—C12—C13	121.9 (2)	C15—N3—Ag1^iii^	116.2 (2)
C12—C13—C14	114.9 (2)	C16—N3—Ag1^iii^	127.6 (2)
C12—C13—H13A	108.5	C14—O1—Ag1^i^	125.2 (2)
C14—C13—H13A	108.5	C14—O2—Ag1	117.96 (17)
			
C6—C1—C2—C3	−0.1 (5)	N1—C7—C12—C13	2.6 (4)
Cl1—C1—C2—C3	−180.0 (3)	C11—C12—C13—C14	−105.3 (3)
C1—C2—C3—C4	−2.0 (5)	C7—C12—C13—C14	75.6 (3)
C2—C3—C4—C5	0.7 (5)	C12—C13—C14—O2	−31.7 (4)
C3—C4—C5—C6	2.7 (5)	C12—C13—C14—O1	149.1 (3)
C3—C4—C5—Cl2	−175.9 (3)	N3—C16—C17—C18	−0.5 (4)
C2—C1—C6—N1	179.0 (3)	C16—C17—C18—N2	0.5 (4)
Cl1—C1—C6—N1	−1.2 (4)	C1—C6—N1—C7	133.5 (3)
C2—C1—C6—C5	3.2 (4)	C5—C6—N1—C7	−51.1 (4)
Cl1—C1—C6—C5	−176.9 (2)	C8—C7—N1—C6	−21.6 (4)
C4—C5—C6—N1	179.8 (3)	C12—C7—N1—C6	157.8 (3)
Cl2—C5—C6—N1	−1.6 (4)	N3—C15—N2—C18	−0.3 (4)
C4—C5—C6—C1	−4.5 (4)	N3—C15—N2—Ag1	−176.8 (2)
Cl2—C5—C6—C1	174.0 (2)	C17—C18—N2—C15	−0.1 (4)
C12—C7—C8—C9	−1.8 (4)	C17—C18—N2—Ag1	176.4 (2)
N1—C7—C8—C9	177.6 (3)	N2—C15—N3—C16	0.3 (4)
C7—C8—C9—C10	−0.2 (4)	N2—C15—N3—Ag1^iii^	173.7 (2)
C8—C9—C10—C11	1.0 (5)	C17—C16—N3—C15	0.2 (4)
C9—C10—C11—C12	0.2 (5)	C17—C16—N3—Ag1^iii^	−172.4 (2)
C10—C11—C12—C7	−2.1 (4)	O2—C14—O1—Ag1^i^	17.1 (4)
C10—C11—C12—C13	178.7 (3)	C13—C14—O1—Ag1^i^	−163.8 (2)
C8—C7—C12—C11	3.0 (4)	O1—C14—O2—Ag1	18.3 (4)
N1—C7—C12—C11	−176.5 (2)	C13—C14—O2—Ag1	−160.8 (2)
C8—C7—C12—C13	−177.9 (2)		

Symmetry codes: (i) −*x*+1, −*y*+1, −*z*+2; (ii) *x*, −*y*+1/2, *z*+1/2; (iii) *x*, −*y*+1/2, *z*−1/2.

### Hydrogen-bond geometry (Å, º)

Cg6 is the centroid of the [please define] ring.

**Table:**

*D*—H···*A*	*D*—H	H···*A*	*D*···*A*	*D*—H···*A*
N1—H1···O2	0.86	2.43	2.971 (3)	122
C16—H16···O1^iv^	0.93	2.51	3.248 (4)	136
C13—H13*B*···*Cg*6^iii^	0.97	3.30	3.983 (3)	129

Symmetry codes: (iii) *x*, −*y*+1/2, *z*−1/2; (iv) *x*, *y*, *z*−1.

## References

[bb2] Addison, A. W., Rao, T. N., Reedijk, J., van Rijn, J. & Verschoor, G. C. (1984). *J. Chem. Soc. Dalton Trans.* pp. 1349–1356.

[bb1] Ali, H. A. & Jabali, B. (2016). *Polyhedron*, **107**, 97–106.

[bb4] Azócar, M., Muñoz, H., Levin, P., Dinamarca, N., Gomez, G., Ibanez, A., Garland, M. T. & Paez, M. A. (2013). *Commun. Inorg. Synth.* **1**, 19–21.

[bb5] Caglar, S., Aydemir, I. E., Adıgüzel, E., Caglar, B., Demir, S. & Büyükgüngör, O. (2013). *Inorg. Chim. Acta*, **408**, 131–138.

[bb6] Caglar, S., Aydemir, I. E., Cankaya, M., Kuzucu, M., Temel, E. & Büyükgüngör, O. (2014). *J. Coord. Chem.* **67**, 969–985.

[bb7] Castellari, C., Feroci, G. & Ottani, S. (1999). *Acta Cryst.* C**55**, 907–910. 10.1107/s0108270199002206 10408083

[bb8] Dimiza, F., Perdih, F., Tangoulis, V., Turel, I., Kessissoglou, D. P. & Psomas, G. (2011). *J. Inorg. Biochem.* **105**, 476–489. 10.1016/j.jinorgbio.2010.08.013 20926136

[bb9] Duffy, C. P., Elliott, C. J., O’Connor, R. A., Heenan, M. M., Coyle, S., Cleary, I. M., Kavanagh, K., Verhaegen, S., O’Loughlin, C. M., NicAmhlaoibh, R. & Clynes, M. (1998). *Eur. J. Cancer*, **34**, 1250–1259. 10.1016/s0959-8049(98)00045-8 9849488

[bb10] Farrugia, L. J. (2012). *J. Appl. Cryst.* **45**, 849–854.

[bb3] Hamamci Alisir, S., Demir, S., Sariboga, B. & Buyukgungor, O. (2015). *J. Coord. Chem.* **68**, 155–168.

[bb11] Iliescu, T., Baia, M. & Kiefer, W. (2004). *Chem. Phys.* **298**, 167–174.

[bb12] Kim, K., Yoon, J., Kim, J. K., Baek, S. J., Eling, T. E., Lee, W. J., Ryu, J., Lee, J. G., Lee, J. & Yoo, J. (2004). *Biochem. Biophys. Res. Commun.* **325**, 1298–1303. 10.1016/j.bbrc.2004.10.176 15555568

[bb13] Kourkoumelis, N., Demertzis, M. A., Kovala-Demertzi, D., Koutsodimou, A. & Moukarika, A. (2004). *Spectrochim. Acta Part A*, **60**, 2253–2259. 10.1016/j.saa.2003.11.027 15249013

[bb14] Kovala-Demertzi, D., Theodorou, A., Demertzis, M. A., Raptopoulou, C. P. & Terzis, A. (1997). *J. Inorg. Biochem.* **65**, 151–157.

[bb15] Njogu, E. M., Omondı, B. & Nyamorı, V. O. (2015). *J. Coord. Chem.* **68**, 3389–3431.

[bb16] Nomiya, K., Takahashi, S. & Noguchi, R. (2000). *J. Chem. Soc. Dalton Trans.* pp. 2091–2097.

[bb17] Olson, L., Whitcomb, D. R., Rajeswaran, M., Blanton, T. N. & Stwertka, B. J. (2006). *Chem. Mater.* **18**, 1667–1674.

[bb18] Pyykkö, P. (1997). *Chem. Rev.* **97**, 597–636. 10.1021/cr940396v 11848883

[bb19] Ribeiro, G., Benadiba, M., Colquhoun, A. & de Oliveira Silva, D. (2008). *Polyhedron*, **27**, 1131–1137.

[bb20] Sheldrick, G. M. (2008). *Acta Cryst.* A**64**, 112–122. 10.1107/S0108767307043930 18156677

[bb21] Sheldrick, G. M. (2015). *Acta Cryst.* C**71**, 3–8.

[bb22] Stoe & Cie (2002). *X-AREA* and *X-RED32*. Stoe & Cie, Darmstadt, Germany.

[bb23] Wang, Z. & Cohen, S. M. (2009). *Chem. Soc. Rev.* **38**, 1315–1329.

[bb24] Weder, J. E., Dillon, C. T., Hambley, T. W., Kennedy, B. J., Lay, P. A., Biffin, J. R., Regtop, H. L. & Davies, N. M. (2002). *Coord. Chem. Rev.* **232**, 95–126.

[bb25] Wu, D. D. & Mak, T. C. W. (1995). *J. Chem. Soc. Dalton Trans.* pp. 2671–2678.

[bb26] Yam, V. W. & Lo, K. (1999). *Chem. Soc. Rev.* **28**, 323–334.

[bb27] Yang, L., Powell, D. R. & Houser, R. P. (2007). *Dalton Trans.* pp. 955–964. 10.1039/b617136b 17308676

[bb28] Zhang, J. P., Huang, X. C. & Chen, X. M. (2009). *Chem. Soc. Rev.* **38**, 2385–2396. 10.1039/b900317g 19623356

[bb29] Zhang, T., Huang, H. Q., Mei, H. X., Wang, D. F., Wang, X., Huang, R. & Zheng, L. (2015). *J. Mol. Struct.* **1100**, 237–244.

