# Supplementary figures and images for: Crystal structure of a mixed-ligand silver(I) complex of the non-steroidal anti-inflammatory drug diclofenac and pyrimidine
Source: Acta Crystallogr E Crystallogr Commun. 2016 Sep 27;72(Pt 10):1475–9. doi: 10.1107/S2056989016014730 (PMC5050780; doi:10.1107/S2056989016014730)

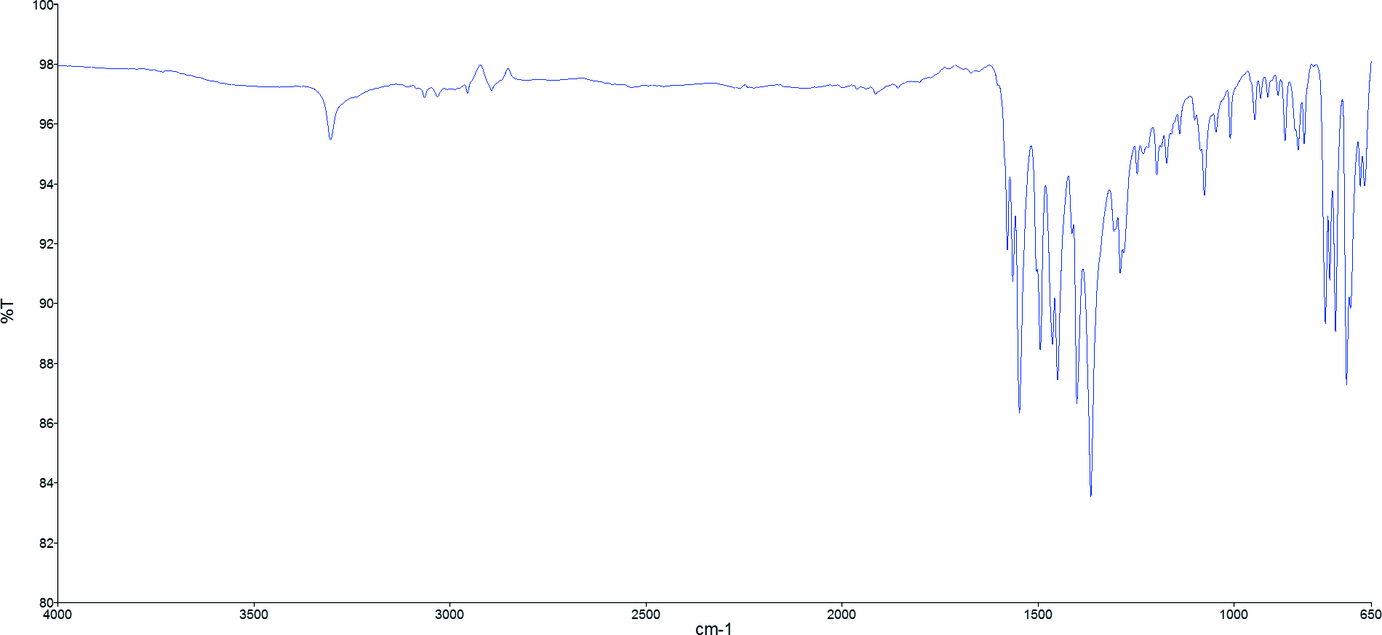

Supplement: Supplementary file 3 [file e-72-01475-sup3.tif]
